# A metric and its derived protein network for evaluation of ortholog database inconsistency

**DOI:** 10.1186/s12859-024-06023-x

**Published:** 2025-01-07

**Authors:** Weijie Yang, Jingsi Ji, Gang Fang

**Affiliations:** 1 NYU-Shanghai, Shanghai, 200120 China; 2 Department of Biology, New York University, New York, NY 10003 USA; 3 Software Engineering Institute, East China Normal University, Shanghai, 200062 China

**Keywords:** Signal Jaccard index (SJI), SJI network, Orthology, Discontinuity in macroevolution

## Abstract

**Background:**

Ortholog prediction, essential for various genomic research areas, faces growing inconsistencies amidst the expanding array of ortholog databases. The common strategy of computing consensus orthologs introduces additional arbitrariness, emphasizing the need to examine the causes of such inconsistencies and identify proteins susceptible to prediction errors.

**Results:**

We introduce the Signal Jaccard Index (SJI), a novel metric rooted in unsupervised genome context clustering, designed to assess protein similarity. Leveraging SJI, we construct a protein network and reveal that peripheral proteins within the network are the primary contributors to inconsistencies in orthology predictions. Furthermore, we show that a protein's degree centrality in the network serves as a strong predictor of its reliability in consensus sets.

**Conclusions:**

We present an objective, unsupervised SJI-based network encompassing all proteins, in which its topological features elucidate ortholog prediction inconsistencies. The degree centrality (DC) effectively identifies error-prone orthology assignments without relying on arbitrary parameters. Notably, DC is stable, unaffected by species selection, and well-suited for ortholog benchmarking. This approach transcends the limitations of universal thresholds, offering a robust and quantitative framework to explore protein evolution and functional relationships.

**Supplementary Information:**

The online version contains supplementary material available at 10.1186/s12859-024-06023-x.

## Background

Our biological understanding, confirmed through laboratory experiments, is derived mainly from a few model organisms. To illustrate, recent statistics from UniProtKB/TrEMBL show that proteins verified 'at the protein level' comprise less than 1‰ of the total 250 million proteins (Release 2023_02, https://www.uniprot.org/). Given its vital role in transferring knowledge from model organisms to many other species [1, 2], orthology is arguably one of the cornerstones of biological studies. Over recent decades, biologists and data scientists have devoted significant effort to identifying orthologs, leading to various computational methods and databases that advance biological research [3]. Nevertheless, consistency issues among these ortholog databases have surfaced [4–6], casting doubt on the accuracy of some studies. For instance, pan-genome studies heavily depend on the differentiation between core and accessory genes, a distinction that forms the basis for subsequent analyses [7].

A recent report shows a mean consistency of around 0.52 [6] among renowned ortholog databases such as eggNOG [8, 9], OrthoFinder [10], and Broccoli [11]. This inconsistency prompts an examination of its origins: are they technical or biological? On the technical side, we could investigate the impact of species selection and arbitrary parameters, such as the e-value or the number of top similar proteins used in the initial ortholog prediction steps. Since these parameters differ across algorithms, they may contribute to the observed inconsistency. Additionally, given the diverse selection pressures on proteins, we should assess the suitability of using uniform parameters for all proteins. Moreover, many algorithms are heuristic [10], raising the question: could the inconsistencies stem from the orthology prediction algorithms themselves? On the biological side, we need to identify which proteins are susceptible to inconsistent ortholog predictions and investigate if they possess common evolutionary traits leading to this inconsistency.

Until these concerns are fully addressed, the common practice is to derive consensus from multiple ortholog predictions [3, 4]. However, generating consensus orthologs introduces subjective elements at several stages. First, the choice of algorithms and databases to compute consensus orthologs is largely discretionary, often dependent on the researcher's preferences or available resources. Second, the consensus threshold—the percentage of algorithms returning the same ortholog predictions—also adds an element of arbitrariness. Finally, because the comparison primarily involves sets of proteins rather than individual pairs, determining whether these sets are "similar enough" to be consolidated into a consensus set involves yet another subjective decision.

Thus, efforts to improve consensus orthologs are gaining significant attention in the biological sciences community. The Quest for Orthologs (QfO) consortium has created the Orthology Benchmark Service, widely viewed as the "gold standard" for orthology prediction [3]. This benchmark includes expertly curated ortholog pairs from sources like SwissTree and TreeFam-A, species phylogenetic trees derived from these orthologs, and functional annotations such as those from Gene Ontology [3]. The establishment of QfO has revolutionized ortholog prediction practices, with all new algorithms now benchmarked against this gold standard, allowing for refined parameter tuning [11]. While QfO signifies a considerable advancement in the quality of ortholog predictions, theoretically, its lack of "true negative" sets [12] might limit its ability to reduce inconsistency as the quantity of ortholog predictions expands [6].

Consequently, we propose a shift from the existing one-class classification methodology to a ranking or scoring framework for benchmarking ortholog predictions. A scoring approach offers two advantages: first, as every ortholog prediction inherently involves a degree of uncertainty, scoring represents a well-established strategy to manage this challenge [12]. Several existing models exemplify this, such as the ratio R in the recently developed Broccoli prediction [11], the duplication consistency score from Ensembl Compara [13], and the InParanoidDB confidence value for inparalogs and seed orthologs [14]. Implementing a scoring system in the gold standard removes exclusive reliance on a single positive label; instead, scores can guide the optimization of parameters, aligning them more closely with the intended purpose. Second, and more importantly, scoring can highlight orthologs with low confidence, which may produce inconsistent results across different algorithms, prompting more detailed analysis.

A scoring system for ortholog confidence should factor in two key dimensions, as in the QfO benchmarking service: phylogeny and functional conservation [3]. Numerous existing scoring systems [11, 13, 15] prioritize phylogeny, offering promising metrics and suggesting potential for their integrated use in a comprehensive scoring system. However, developing an objective metric for the other dimension, protein functional conservation, remains a challenge. Current methods, which rely primarily on Gene Ontology or Enzyme Commission conservation tests, essentially depend on experimental results from a few selected model organisms and protein sequence alignment. A common practice of using sequence conservation to gauge function has inherent shortcomings: different proteins are subjected to varying selection pressures, which renders sequence similarities an ineffective measure for evaluating ortholog functional conservation across protein families. Given the routine use of orthologs for genome annotation and functional comparative genomics, protein function conservation has been integral to ortholog prediction since the advent of the BBH (Bidirectional Best Hit) and COG algorithms [16]. BBH, as the first algorithm to incorporate genome context in ortholog prediction, posits that the mutually most similar protein pairs in different organisms likely share the same function [5, 16]. However, this assumption can be disrupted by evolutionary phenomena like sub-functionalization and neo-functionalization [5], suggesting that the most similar genes may not necessarily perform identical functions. Furthermore, gene duplication can compromise the integrity of BBH ortholog groups [5]. Although mitigation strategies exist, such as PANTHER's 'least diverged orthologs (LDO)' approach [17] and the OMA Group's methodology [18], they often overlook inparalogs, which play a crucial role in projecting biological function across species [19, 20]. In response, we propose a protein network-based scoring system that facilitates cross-family evaluations of protein sequence and functional conservation, and, crucially, incorporates inparalogs into the scoring metric.

We constructed our protein network based on a metric inspired by studies on protein fitness. Protein fitness represents a protein's functional performance that is not solely a product of its sequence but is also influenced by the overall genomic context and environmental factors [21]. Recent studies highlight that the evolution of amino acids within protein sequences is not an independent process, especially when considering protein fitness [22, 23]. This concept of interconnectedness in amino acid mutations inspired our hypothesis of 'discontinuous' evolution in protein sequences: within an ortholog group (OG), amino acid substitutions can accumulate without altering the OG's function, implying continuous evolution toward a common fitness peak; conversely, the emergence of new OGs, driven by substantial amino acid mutations, fosters functional divergence, which might be represented as separate peaks on a fitness landscape. This prompts the question: Can discontinuous evolutionary patterns between proteins be detected solely through comparisons of protein repertoires and sequence data across species? Our hypothesis suggests that these discontinuities, analogous to valleys between fitness peaks, can be identified by analyzing protein repertoire and sequence data, without direct fitness measurements.

Accordingly, we test this hypothesis of discontinuous protein evolution and propose a network-based method to include inparalogs in high-confidence ortholog groups (OGs), comparable to PANTHER's LDO [17] and OMA Groups [18]. In our approach, we first apply an unsupervised spectral clustering algorithm to identify ortholog candidates, named "signals," which can accommodate varying selection pressures on different proteins. Subsequently, we devise a Signal Jaccard Index (SJI) metric to gauge protein similarity, independent of predetermined arbitrary parameters. Utilizing SJI allows us to quantify the similarity between each pair of proteins, laying the foundation for constructing our derived protein network. In this network, each protein's degree centrality (DC), representing the sum of all connected edge weights measured by SJI, becomes a pivotal component of our proposed scoring system. Our method can discern the selection pressures influencing varying protein sequence similarity and highlight the main inconsistencies in existing ortholog databases: fast-evolving large ortholog groups and proteins that are part of small ortholog groups. These proteins that challenge ortholog predictions typically appear in peripheral regions of our derived protein network, signified by low DC values.

Lastly, we show that DC serves as a very efficient score for refining consensus orthologs, the intersections of predictions from various algorithms or databases. And using DC to score protein fitness bypasses the need for complex operations to identify intersections from multiple ortholog databases. It also eliminates potential arbitrariness introduced by the choice of parameters in calculating database intersections. Furthermore, we elaborate on inferences drawn from the topology of the derived network. By introducing protein DC, we aim to stimulate discussions on the creation of a more objective orthology scoring system.

## Methods

### Eukaryotic proteomes and selection of bacterial proteomes

The Quest for Orthologs (QfO) Benchmarking service, a community-driven web service, was our primary source for eukaryotic proteomes. This platform is dedicated to facilitating the benchmarking of ortholog prediction methods. From QfO, we procured the 2021 version of 48 eukaryotic reference proteomes [24].

We recognized that QfO does not include an extensive collection of bacterial genomes, so we sought additional resources. We turned to the EMBL-EBI to download a broad range of bacterial genomes. However, we noticed a pronounced bias in the bacterial species that had been fully sequenced, with a preponderance of pathogens and model organisms represented.

To alleviate this bias and ensure an equitable representation of bacterial diversity, we employed a phylogenetic tree from Greengenes, based on 16S rRNA data from 2013 [25]. We partitioned this tree into 400 clades, ensuring that the evolutionary distances amongst these clades were approximately equivalent. We selected one genome from each of these clades, resulting in a collection of 367 bacterial proteomes. Notably, some clades did not include fully sequenced genomes.

In summary, we carefully selected a diverse range of bacterial and eukaryotic proteomes used in our study to minimize bias and maximize the representation of evolutionary history. All these proteomes are available at our dedicated online repository, www.protdc.org.

### 2D plot and spectral clustering to detect signals

In our study, we treated each protein as a seed. Using the OPSCAN tool [26], we identified the top 10 and 50 most similar proteins from bacterial and eukaryotic proteomes, respectively. OPSCAN computes full-length global protein similarity via the NWS algorithm [26], helping to bypass chimeric protein issues associated with local alignment similarities. To address the complication of multiple protein domains, we selected top similar proteins considering a length difference ratio (longer to shorter protein) of less than 1.5.

**Fig. 1:**
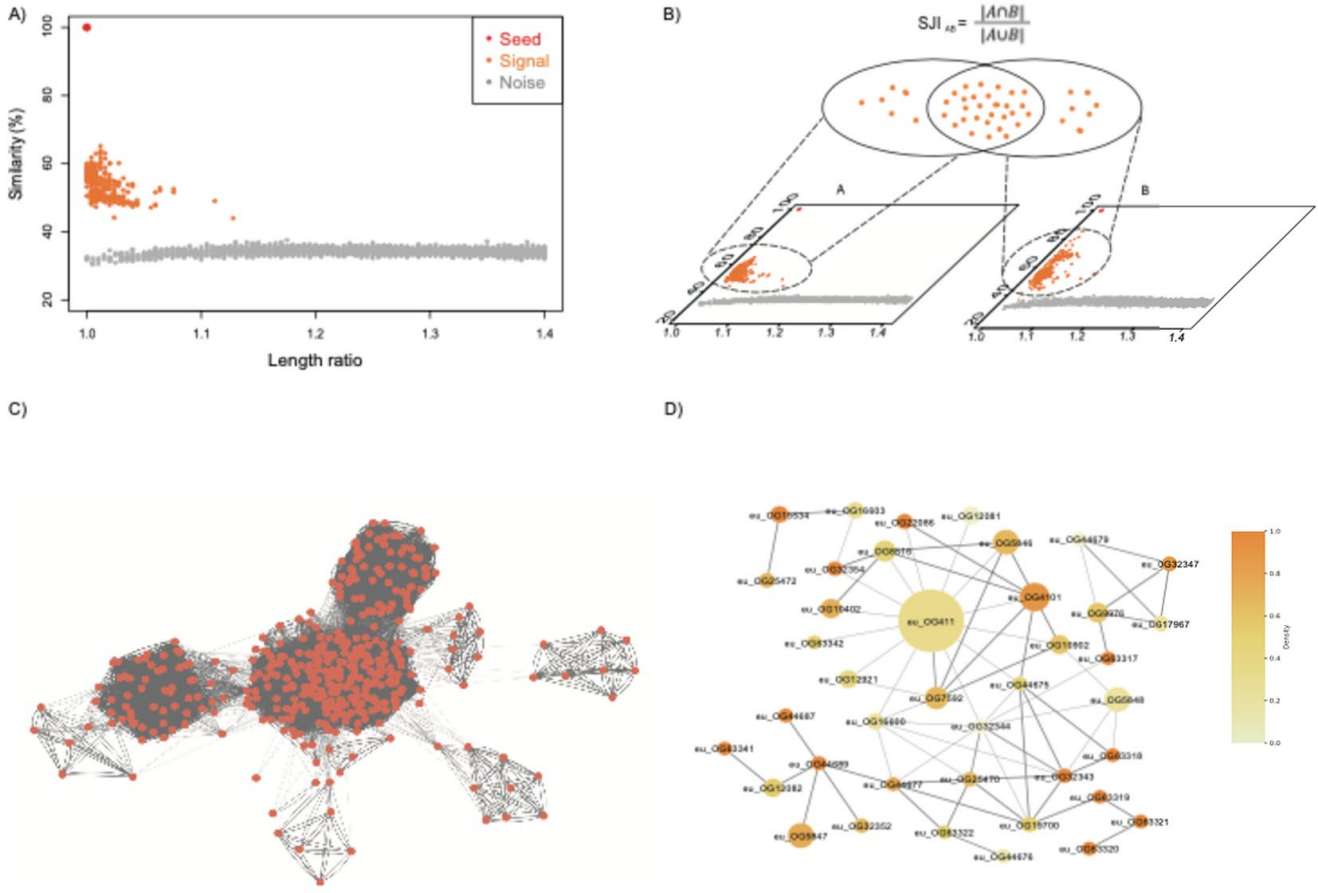
Overview of the workflow. **A** 2D plot depicting the relative coordinates of top similar proteins from various proteomes compared to a seed protein. The x-axis represents the length ratio between the proteins and the seed, calculated by dividing the longer length by the shorter. All proteins displayed on the 2D plot have a length ratio of less than 1.5. The y-axis represents the global sequence similarity between the proteins and the seed, calculated using OPSCAN. A spectral clustering algorithm separates the signals (shown in orange) from the noises (shown in grey). **B** Schematic diagram illustrating the Signal Jaccard Index (SJI). The SJI quantifies the overlap of signals between two seeds, determined by the size of their signal set intersection divided by the size of their signal set union. **C** Partial view of the resulting network. The nodes represent seeds, and the edges are weighted using the SJI. This network undergoes the Louvain algorithm for ortholog group detection. **D** Partial view of the network after detecting OGs. Nodes represent OGs, with sizes proportional to the number of proteins each contains. Node colors indicate the density, reflecting connectivity among proteins within each OG. Node labels correspond to OG IDs used in our ProtDC database (www.protdc.org). Edges between OGs show their connections from the original network, weighted by the normalized sum of the Signal Jaccard Index (SJI) across OGs

We then plotted each seed and its similar proteins on a 2D plot (Fig. 1A), with the x-axis representing the protein length ratio (ranging from 1 to 1.5), and the y-axis showing protein sequence similarity. In each 2D plot, we utilized a spectral clustering algorithm [27] to distinguish signals from noise. The primary steps of this algorithm are as follows:

1. *Input*: Two-dimensional points of size *n*, where *n* represents the number of proteins.
2. *Zero Mean Normalization*: Conduct normalization on both dimensions.
3. *Graph Construction*: Develop a similarity graph *G* by calculating the Euclidean distance between each point, and generate the adjacency matrix *A* and degree matrix *D*.
4. *Laplacian Matrix*: Compute the random walk normalized Laplacian matrix *L*_*rw*_ = *D*^*−1*^*L* = *I-D*^*−1*^*A.*
5. *Cluster Determination*: Ascertain the number of clusters *k* that maximizes the eigengap.
6. *Eigenvalue and Eigenvectors*: Identify the smallest *k* eigenvalues and corresponding eigenvectors *x*_*1*_*, …, x*_*k*_ of *L*_*rw*_.
7. *Matrix Creation*: Denote *U* as a matrix containing eigenvectors *x*_*1*_*, …, x*_*k*_ as columns, its size is *n x k*, and n represents the number of points.
8. *K-Means Partition*: Implement the K-Means algorithm on matrix *U* to segregate the data points into *k* clusters *C*_*1*_*, …, C*_*k*_.
9. *Output*: Clusters *C*_*1*_*, …, C*_*k*_.

Our setup ensures that the largest cluster, characterized by the highest density, represents noise. Occasionally, a few smaller clusters emerge to the right of the largest cluster. These clusters, owing to their excessive protein length difference, are also classified as noise. In contrast, all other clusters that are noticeably separated from these noise clusters are considered signals.

**Fig. 2:**
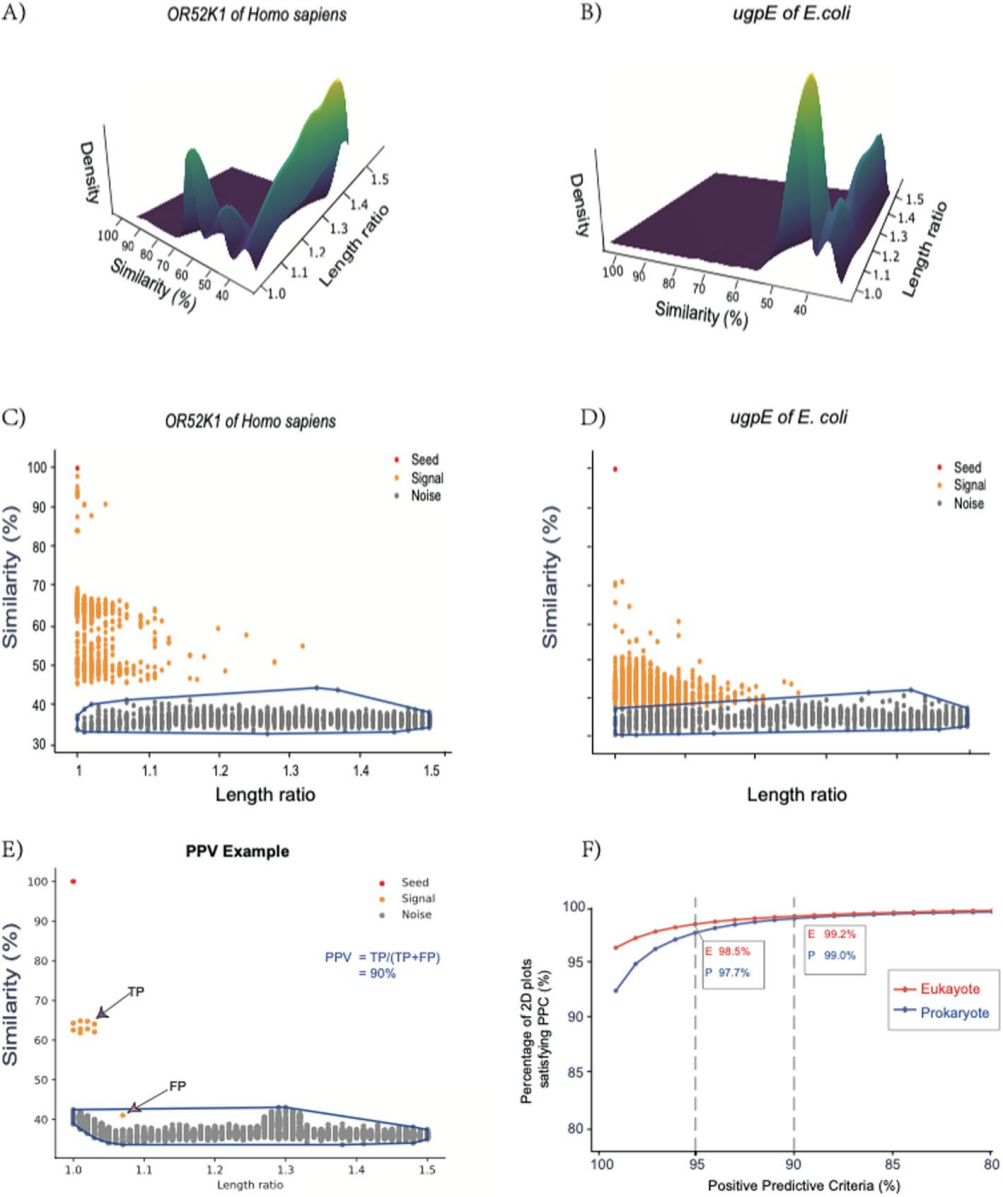
Discontinuous protein sequence evolution across OGs. **A** Density plot illustrating the density of signals and noises for the human olfactory receptor OR52K1. Density is computed based on kernel estimation using the kde2d R package [52]. **B** Another density plot example featuring ugpE, a transmembrane component of the *E. coli* ABC transporter. **C** Convex hull outlining the noises of OR52K1, drawn using the qhull algorithm from the SciPy package. **D** Convex hull outlining the noise associated with ugpE. **E** Schematic figure illustrating the definitions of True Positive (TP), False Positive (FP), and Positive Predictive Value (PPV). **F** Evaluation of signal precision rate. This diagram summarizes the signal PPV for all 2 million seeds. The signal PPV on each 2D plot is defined as the percentage of signals appearing above the noise-convex hull. The x-axis represents the PPV critical values, and the y-axis represents the percentage of 2D plots meeting each PPV criterion

To evaluate the performance of our clustering, we outlined a convex hull around the noise. We defined signal Positive Predictive Value (PPV) as the proportion of signal proteins that lie above this noise hull (Fig. 2E). The convex hull was designed using the qhull algorithm from the SciPy Python package [28, 29]. Additionally, we scrutinized the BLAST E-values between seed-signal and seed-noise pairs.

### Derived protein similarity network

To distinguish signals from noise, we considered the biological information encompassed within the entire proteome. Each protein serves as a seed, and seeds belonging to the same fitness landscape peak should have a better signal overlap. As such, we utilized the Jaccard similarity coefficient as an indicator to measure the functional similarity between seeds. As depicted in Fig. 1B, we calculated the Signal Jaccard Index (SJI), defined as the intersection of two signal sets divided by their union. The SJI measures functional similarity and serves as the edge weight between all pairs of seeds, thereby enabling the automatic construction of a function-oriented network that connects all proteins (Fig. 1C). 

### Ortholog group detection

We identified ortholog groups as communities in the network using the Louvain algorithm [30], designed to maximize community modularity [31]. Due to inevitable low-weight edges, our initial network was extensive, which necessitated multiple applications of the Louvain algorithm. To address this, we employed a stepwise zoom-in strategy until communities reached a density threshold. The density "D" of a community considers the edge weights and is defined by the following formula:

$${\text{D}} = \frac{{2 \cdot \Sigma_{u \in N,v \in N, u \ne v} W\left( {u,v} \right)}}{{\left| N \right| \cdot \left| {N - 1} \right|}}$$ *.*

Here, N represents the number of nodes in a community, and *W* represents the weight between nodes *u* and* v*. Our ortholog group detection procedure is as follows:

1. Input: A weighted protein similarity network.
2. Compute the network density *D* using the formula above. If *D* exceeds a certain threshold, halt the network cutting process and designate the current network as an Ortholog Group (OG). If not, proceed to step 3.
3. Apply the Louvain algorithm to dissect the current network into sub-networks.
4. For each resulting sub-network, update the edge weights considering only the nodes (seeds) included in that sub-network.
5. Repeat steps 2–4 for each sub-network.

### Benchmark orthology databases

To assess our OGs, we submitted our ortholog pairs to the QfO Benchmarking service. We first built phylogenetic trees following the eggnog41 workflow suggested by eggNOG [3] using the ETE toolkit v3 [32] for our OGs (including the set with density ≥ 0.25), and then obtained ortholog pairs via the species overlap algorithm [33]. We limited our comparison to eukaryotic OGs due to differences in bacterial proteomes, and submitted our results to benchmark against QfO's TreeFam-A ortholog pairs.

In addition, as our method’s metric and network emphasize biological constraints in the genome context, we also compared our OGs to those from TreeFam, eggNOG, Broccoli, OrthoFinder, OrthoDB, and SonicParanoid using the Adjusted Rand Index (ARI) and Adjusted Mutual Information (AMI), two standard measures for clustering comparison. While ARI gauges consistency based on pair counts, AMI utilizes Shannon information [34].

## Results

### Delineating signal from noise: an unsupervised method for 2D protein similarity plot analysis

Previous studies on the protein fitness landscape and sequence evolution [22, 23, 35] inspired our current work. One study suggested that proteins could elevate fitness by undergoing extensive amino acid mutations [35]. Another study demonstrated strong correlations between mutations at different amino acid sites, limiting the number of independent evolutionary paths from a given protein sequence [22]. Additionally, a machine learning model developed a low-dimensional space, elucidating the relationship between protein sequence evolution and fitness stability [23]. This low-dimensional space suggests that amino acid mutations are interdependent [23]. The significance of these correlations was also evident in protein sequence diversity [36]. These findings suggest a discontinuous protein sequence evolution hypothesis: if we view Ortholog Groups (OGs) from a protein functional adaptation standpoint, OGs should appear as distinct "peaks" on the protein fitness landscape. Each peak encapsulates critical, mutually constrained amino acid positions essential to protein function. When adapting to new functions, proteins undergo significant mutations, thus creating another peak with a distinct set of mutually constrained positions. Other positions, subject to less restrictive, independent mutations, result in continuous sequence variation within the OG.

Drawing upon these insights, we devised a simple two-dimensional (2D) plot as an economical means to test this hypothesis. This plot's axes are protein sequence similarity and protein length difference. We utilized a bacterial protein A as a "seed" for illustration in Fig. 1A. We selected the top 10 proteins most similar to seed A from each of the 367 chosen bacterial genomes (Methods), resulting in a total of 3670 proteins. We chose the top 10 to encompass both ortholog candidates ("signal") and a "sufficient excess" of distant homologs ("noise") of seed A in one plot, given the rarity of finding 10 or more inparalogs from the same species for a bacterial ortholog group (according to the data from InParanoid 8, the average count of inparalogs per species is 1.41 [37]). Considering that the entire genome context or protein repertoire encodes all functions within a species, our focus was on the top 10 most similar proteins from each prokaryotic species. This selection is based on the premise that functionally consistent orthologs, if present, are assured to be among these top proteins. The number 10 is considered sufficiently large for bacterial genomes to ensure that the majority of these proteins—totaling 3670 across various species in this demonstration—have undergone significant functional evolutionary events. Consequently, these proteins are likely to belong to distinct fitness peaks, thus forming separate OGs. This design allows us to examine whether there is a "valley" that separates "signal" from "noise" on our 2D plot. The discontinuous hypothesis posits the "valley" as evidence of distinct evolutionary paths. We separately applied this test to prokaryotic and eukaryotic proteins. Due to the complexity and high gene duplication rate of eukaryotic genomes [38], we expanded our selection to the top 50 for eukaryotic proteins (Methods). Given the dose imbalance effect in eukaryotes [39] and based on a survey of existing ortholog databases, the top 50 ensures that eukaryotic 2D plots contain sufficient noise.

To illustrate the distinction between signal and noise, we spotlight two particularly challenging proteins in Fig. 2AB: the *E. coli* ugpE and the human olfactory receptor 52K1. Each represents one of the most expansive and rapidly evolving protein families in their respective domains, prokaryotes and eukaryotes [40, 41]. We have generated 2D plots for all 1,231,088 bacterial and 889,930 eukaryotic seed proteins, demonstrating this demarcation. In most plots, signal and noise regions do not share a border, presenting a "gap" rather than a "valley," indicating a clear distinction. Additional illustrations of this gap are shown in Supp. Figure 1. All these 2 million plots are publicly available at www.protdc.org.

Beyond example observations, we conducted statistical validation. We applied a spectral clustering algorithm (Methods), successfully identifying the valleys that separate signals from noise for the two challenging seed proteins discussed earlier (Fig. 2CD). This spectral clustering approach is unsupervised, focusing solely on the data's inherent structure without reliance on preset parameters. It generates clusters based on the density of data points on the 2D plot. Importantly, a protein's relative proximity to the seed on this plot does not influence the clustering (Methods). As a result, the noise proteins congregate in an irregular area at the lower part of the 2D plot (Fig. 2C–E). To assess signal reliability, we draw a convex hull enclosing this irregular noise area; signals falling within this hull are considered "false positives" due to their lower sequence similarity or greater protein length difference relative to certain noise proteins (Fig. 2E).

By delineating a convex boundary for the noise data points, we can estimate the Positive Predictive Value (PPV) of the predicted signals. As depicted in Fig. 2E, true positives (TP) are signals located above the noise convex hull, while false positives (FP) are signals found within this hull. We define the signal's PPV as TP/(TP + FP) (Fig. 2E). Remarkably, more than 90% of these 2 million plots achieved a PPV of 100% (Fig. 2F), with no signal data points falling within the noise convex hull. Furthermore, an impressive 98.5% of 2D plots from eukaryotes and 97.7% from prokaryotes maintained a PPV exceeding 95% (Fig. 2F).

We additionally assessed the BLAST E-value of seed-signal pairs. The E-values for these pairs were strikingly significant, with over 97% (comprising 208 million prokaryotic and 47 million eukaryotic pairs) exhibiting E-values less than 10^-6. Interestingly, there were also 150 million prokaryotic and 46 million eukaryotic seed-noise pairs presenting strong E-values less than 10^-6. Given the evident distinction between signals and noise (Supp. Figure 1), these compelling E-values beneath the valleys suggest caution against the exclusive reliance on E-values or arbitrary similarity cutoffs in the selection of candidate orthologs. This is especially pertinent as these "significant" noise data points are intertwined with and inseparable from other "less-significant" noise data points. It's important to note that the Y-axis on our 2D plots is based on the score of protein full-length global alignment (Methods), while the BLAST E-value is determined by local alignment.

In conclusion, our results substantiate the hypothesis that protein sequence evolution is discontinuous as Ortholog Groups (OGs) diversify and new functions arise. The discernible demarcation between signal and noise, coupled with high signal PPV, evident across all 2D plots (Fig. 2F), underscores this notion. It's worth noting that this hypothesis pertains to protein evolution across species—a macroevolutionary context. Instances where single point mutations lead to substantial functional changes, such as sickle cell anemia, fall within a microevolutionary context, operating on a different evolutionary scale. While this hypothesis presents a general trend applicable to most protein evolution, rare exceptional cases may exist where proteins share the same function despite low similarity.

### Introduction of the SJI metric and its application in building a protein network

By discerning signals from noise, we could evaluate the overlap of signals originating from different seed proteins. If two seeds belong to the same OG, their respective signal sets are expected to overlap significantly. This high degree of overlap also indicates a high level of functional similarity between the seed proteins. We quantified this overlap using the Signal Jaccard Index (SJI), as illustrated in Fig. 1B. SJI evaluates protein similarity using the Jaccard index, which is calculated by dividing the intersection by the union of the 'signals' for each pair of proteins. The calculated SJIs for prokaryotic and eukaryotic seeds are summarized in Supp. Figure 2AB. Notably, for 208 million pairs of prokaryotic seeds, the median SJI was 0.70, while for 48 million eukaryotic seed pairs, the median SJI was 0.63.

We view SJI as an enhancement of the BBH approach. While BBH identifies protein pairs based on a 1:1 mutual best match, it overlooks proteins resulting from recent duplication that have the same function or proteins that underwent sub-/neo-functionalization and now belong to different OGs. In contrast, SJI can account for these nuances. A distinct boundary between signals and noise emerges when sufficient noise is incorporated into the 2D plot. Proteins derived from recent duplication that retain the same fitness will likely fall within the signal set. Conversely, sub-/neo-functionalized proteins—provided that they're not evolutionary transients and their functional diversification events occurred long ago—should be found within the noise group.

Having computed the SJI values for all seed proteins, we proceeded to construct a network linking all proteins. Networks are potent tools for integrating and refining biological information, often filtering out spurious low-SJI connections, thus yielding reliable biological insights from their topology [42]. Proteins under strong evolutionary pressure, consistently exhibiting high SJI values, are categorized into communities. In contrast, unique proteins with multifunctionality due to domain variation may demonstrate high betweenness centrality. Supp. Figure 2C–H offers an overview of the initial network, presenting all proteins as nodes and SJIs as weighted edges.

### Predicting ortholog groups

Low SJI between some proteins is unavoidable, leading to an extensive initial network encompassing nearly all proteins. However, the evident community structure within the network enables us to apply a stepwise clustering strategy to delineate OGs (Methods). We utilized a weighted community density as the cluster convergence threshold (Methods), the only parameter users need to decide on in this work, which influences the OG size. We present two sets of OGs based on two thresholds: density ≥ 0.25 and density ≥ 0.1, in our online database (www.protdc.org). The higher threshold (density ≥ 0.25) implies that, on average, > 50% of nodes within an OG are connected, with edge weights (SJIs) > 0.5. This stringent threshold yielded 73,503 eukaryotic OGs and 72,541 prokaryotic OGs. Lowering this threshold permits the inclusion of more distant orthologs into the OG. The lower threshold (density ≥ 0.1) resulted in 53,418 eukaryotic OGs and 50,939 prokaryotic OGs.

Since our OGs stem from network communities, we assigned a degree centrality (DC) to each protein. DC, normalized by OG size, reflects the extent of overlap between a protein's signal set and its neighboring proteins. We performed a bootstrap analysis to assess the network structure's stability and the reliability of protein DCs. In this process, we randomly removed 10% of proteins, followed the same procedure and threshold to ascertain OGs and protein DC, and repeated this process 1,000 times. Comparing the bootstrap OG with the original results using the Adjusted Rand Index (ARI) (Methods) yielded an average ARI of 0.93; moreover, the average correlation coefficient between the bootstrap DC and the original DC was 0.85. These results demonstrate a stable network structure and protein degree centrality, with Supp. Figure 3 providing detailed insights into the bootstrap analysis.

All OGs are readily available for download at www.protdc.org.

### Benchmarking predicted ortholog groups against QfO service

The Quest for Orthologs (QfO) Benchmark Service is an orthology prediction quality assessment service developed by a consortium of experienced scientists [3]. This service is extensively used to measure the agreement between a developer's ortholog pairs and curated QfO references derived from SwissTree and TreeFam-A [3]. To construct our eukaryotic OGs, we used the QfO 2021 release comprising 48 eukaryotic proteomes. However, QfO does not provide a sufficient number of prokaryotic proteomes. To mitigate this, we obtained 367 bacterial species, evenly distributed on the Greengenes phylogenetic tree [25], from EMBL-EBI (Methods). As a result, we could only benchmark our eukaryotic OGs against TreeFam-A [43]. Detailed benchmark results are presented in Supp. Figure 4. We achieved a high Positive Predictive Value (PPV) of 0.952, but only a modest True Positive Rate (TPR) of 0.556 for our OGs with density ≥ 0.1 (PPV = 0.956, TPR = 0.518 for OGs with density ≥ 0.25). Hereafter, "our" OGs refer to OG sets obtained using density ≥ 0.1 unless otherwise specified. For perspective, some other ortholog databases in Supp. Figure 4 demonstrated high TPRs, ranging from 0.656 to 0.675. However, we noted that TreeFam-A does include some seed-noise ortholog pairs, potentially explaining our lower recall. This occurrence is despite the visible gaps between signals and noises, as showcased in Supp. Figure 1. Regrettably, we could not access the complete TreeFam-A ortholog pairs from QfO. Instead, we downloaded four sets of ortholog pairs with the highest TPR (0.656–0.675) from QfO, discovering that 36–56% of these ortholog pairs were seed-noise pairs (Supp. Table 1). Interestingly, when examining the intersections of these four sets of ortholog pairs, we observe a reduction in seed-noise pairs to 21%, which correspondingly increases seed-signal pairs to 79% (Supp. Table 1). This significant reduction in seed-noise pairs, along with the high PPV (close to 1) returned from the QfO Benchmark Service, suggests a lower reliability in treating seed-noise pairs as ortholog pairs.

The modest TPR may also stem from the inherent differences between TreeFam-A and our OGs. Our algorithm does not incorporate phylogenetic information, which likely contributes to the modest TPR observed in comparisons with TreeFam-A. Similar to our method, the BBH approach prioritizes functional conservation over phylogenetic relationships. Algorithms derived from BBH, such as OMA Groups and Panther's LDO, also do not rely on phylogenetic trees. As illustrated in Supp. Figure 4, OMA Groups, Panther LDO, and our algorithm all exhibit relatively low TPRs. However, our algorithm outperforms both OMA Groups and Panther LDO in terms of TPR, primarily due to the inclusion of inparalogs. Notably, our algorithm achieved a high TPR of 0.937 using the VGNC benchmark, which was recently included in the QFO benchmarks [12]; moreover, BBH-derived algorithms outperform nearly all other algorithms in the VGNC benchmark, as shown in Supp. Figure 4.

### Inconsistency in existing ortholog predictions predominantly attributed to small and large OGs

Using eukaryotic OGs derived from our protein network, we investigated factors contributing to inconsistent prediction results across different algorithms. We examined the size of our OGs. These OGs range from the smallest ones, containing just one protein, to the largest ones, comprising over 1,000 proteins. After calculating the modularity of the initial protein network, we found a high modularity score of 0.99, suggesting strong intra-OG protein connections and weak inter-OG connections in our initially derived network. Hence, from a network structure perspective, midsize to large OGs form the cores of the network, while small OGs make up its peripheries.

We next examined how OG size influences consistency among several ortholog databases, including TreeFam [43], Broccoli [11], OrthoFinder [10], OrthoDB [44], eggNOG [8], and SonicParanoid [45]. These databases employ diverse algorithms for orthology prediction. TreeFam, known for its high-quality predictions, serves as the curated reference in QfO. Broccoli and OrthoFinder use phylogeny-based methods for deriving OGs, while eggNOG and OrthoDB are based on clustering algorithms. We also included SonicParanoid, given its genealogical relationship with InParanoid [14], an algorithm that identifies orthologs by giving more weight to protein functional similarity.

**Fig. 3:**
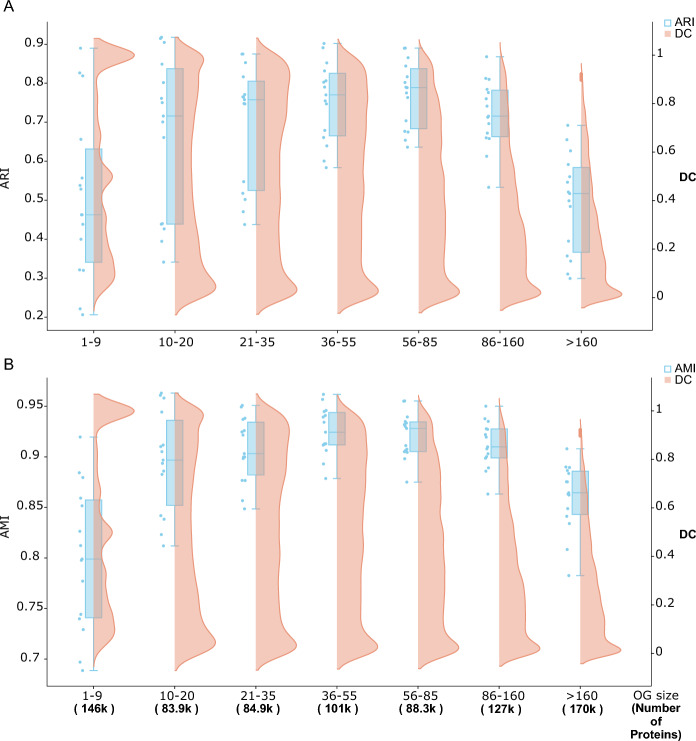
Impact of OG size on the consistency of eukaryotic ortholog predictions. **A** OG Size vs. DC Distribution vs. ARI. Eukaryotic OGs were grouped based on size, represented on the x-axis. The leftmost bin contains around 146 K proteins from OGs of 1–9 proteins, whereas the rightmost bin holds 170 K proteins from OGs with at least 160 proteins. Intermediate bins accommodate around 80–100 K proteins. The orange curve illustrates the normalized DC distribution, with a notable bias towards larger DC in smaller OGs (size <  = 9). The blue boxplot depicts ARI, indicating the concordance between TreeFam, eggNOG, Broccoli, OrthoDB, and OrthoFinder. Detailed numerical data for the blue boxplot are provided in Supp. Table 2. **B** OG size vs. Distribution of DC vs. AMI is shown in this panel

To evaluate the effect of OG size on algorithm consistency, we grouped proteins into seven bins according to their OG sizes (Fig. 3), with each bin containing approximately 100 K proteins. We then assessed pairwise consistency among the six algorithms using both ARI and AMI (Methods). As shown in Fig. 3, there is an increasing trend in ARI and AMI values from the first bin to the fifth as the OG size increases from 1 to 85. The first bin shows the poorest consistency, containing proteins from the network's peripheries. This indicates that gleaning biological insights from peripheral network structures may challenge various algorithms. Researchers should thus acknowledge that different databases may produce unique gene sets, especially when studying rare, species-specific, or pan-genomic accessory genes.

In contrast, consistency in ortholog predictions for proteins in mid-sized OGs seems higher across various algorithms, likely because these OGs are supported by a group of proteins with significant SJI. Notably, Fig. 3 also shows a decrease in the consistency of proteins in the largest OGs (size > 160). This suggests that rapidly evolving proteins might cause inconsistencies, potentially due to their increased likelihood of diverging significantly in structure or function, leading to differing predictions by various algorithms.

### Degree centrality: a scoring system to refine high confidence orthologs

Our study demonstrates that the largest OGs also contribute to the inconsistency observed in ortholog predictions. To counter this issue, we investigated the utility of Degree Centrality (DC) as a scoring metric to refine the selection of consensus, or "high-quality", orthologs.

**Fig. 4:**
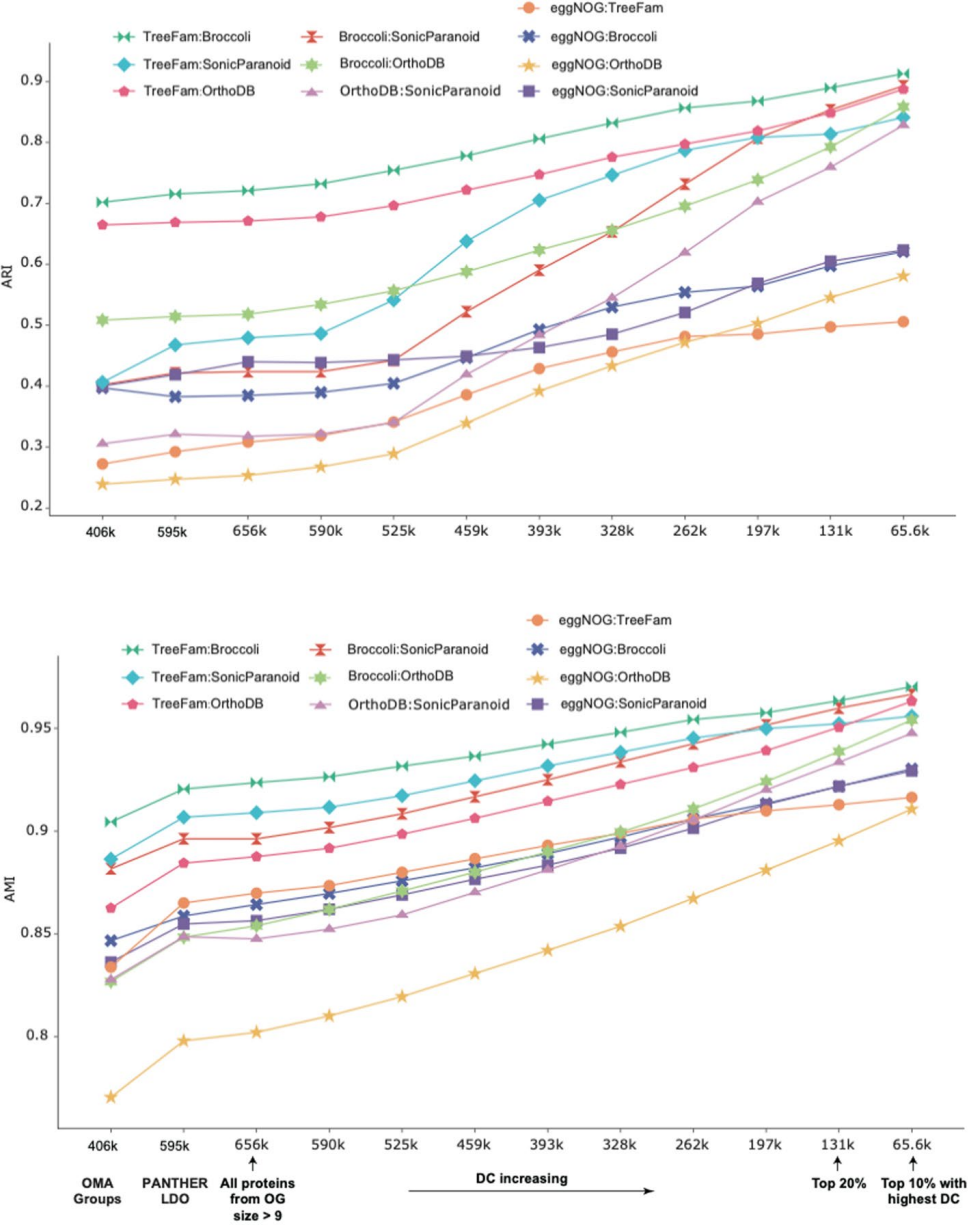
Refining consensus orthologs using Degree Centrality (DC) as a scoring metric. This figure presents an analysis of 656 K eukaryotic proteins derived from all OGs with a size greater than nine. These proteins were divided into ten bins. Each bin, moving from left to right, progressively excludes the 10% of proteins with the lowest DC values. For instance, the first bin includes all 656 K proteins, the subsequent one contains 590 K, and the rightmost bin holds 65.6 K proteins with the highest DC scores. As control groups, we incorporated 406 K proteins from the OMA Groups and 595 K Least Diverged Orthologs (LDO) from PANTHER. All of these proteins come from the 48 eukaryotic reference proteomes. The OMA Groups and PANTHER's LDO orthologs are considered as “highly reliable orthologs”, and they establish a baseline for the subsequent Adjusted Rand Index (ARI) and Adjusted Mutual Information (AMI) comparisons. The concordance among ortholog databases within each bin was evaluated using ARI and AMI. We observed a consistent increase in concordance between pairwise ortholog databases as we moved across the bins, starting from the two control groups

We initiated this process by assembling proteins from OGs with a size greater than 9, thereby excluding the first bin depicted in Fig. 3 due to its contribution to inconsistency. This selection process yielded a dataset of 656 K eukaryotic proteins. Following a DC-based sorting of these proteins, we categorized them into ten overlapping groups as shown on the x-axis of Fig. 4. Starting with the first group, which contained all 656 K proteins, each subsequent group excluded the lowest 10% of proteins based on their DC scores from the previous group. This process culminated in the rightmost group, which included the top 10% of proteins featuring the highest DC scores.

In Fig. 4, we also incorporated control groups consisting of PANTHER's Least Diverged Orthologs (LDO) and the OMA Groups for comparative purposes. Compared to the LDO (595 K proteins) and OMA Groups (406 K proteins), our OGs—encompassing 656 K proteins—exhibit a 10% to 35% greater protein inclusion, attributed predominantly to the incorporation of inparalogs. Over 92% of our OGs portrayed in Fig. 4 contain at least one inparalog. While both LDO and OMA Groups are regarded as "highly reliable" ortholog controls [17, 18], they do not accommodate inparalogs, a characteristic distinguishing our OGs. This distinction is validated by the QfO benchmarking results (Supp. Figure 4), which show that our orthologs exhibit precision comparable to the controls.

The curves in Fig. 4 illustrate the trends in ARI and AMI between ortholog databases across the ten groups, alongside the two control groups. Consistency between all database pairs, with the exception of OrthoFinder (Supp. Figure 5), increases monotonically as protein DC increases. For instance, on the far right of Fig. 4, where 90% of proteins with low DC scores have been excluded, the ARI between TreeFam and all other databases, except for eggNOG, rises to between 0.85 and 0.90. A recent study [6] found such high ARI values only in 125 human-curated OGs. However, as shown in Fig. 4 and Supp. Table 3, these high ARI values are reached with more than 1600 OGs covering between 20 and 45 K proteins. Supp. Table 3 also provides the detailed values corresponding to Fig. 4.

Supp. Figure 6 showcases the intersections between the representative ortholog databases mentioned earlier, emphasizing the modest agreement in their ortholog predictions and highlighting the lack of certainty when relying on intersections for consensus orthologs. Notably, we find a slight positive correlation between degree centrality (DC) and the degree of ortholog overlap (Supp. Figure 6).

Our findings illustrate that applying degree centrality (DC) as a scoring metric for refining ortholog groups derived from different algorithms can yield "high-quality" datasets on par with manually curated ortholog groups. This is particularly useful when studying rapidly evolving large protein families, where assessing each member's DC scores is advisable. Such assessment helps to distinguish between core members, which represent high-confidence orthologs, and peripheral members, which necessitate further scrutiny. This distinction is crucial as different algorithms may categorize these peripheral members into inconsistent ortholog groups. Given that consensus across different ortholog databases is often seen as a robust indicator of orthology, the use of DC also helps alleviate the influence of subjective decisions, such as concerns about which ortholog databases to utilize or determining the degree of overlap needed to designate consensus orthologs.

## Discussion

Our work here primarily deals with the challenges posed by the inconsistencies in ortholog predictions. We attributed these inconsistencies to peripheral nodes in the derived protein network, marked by small ortholog groups (OGs) and proteins with low degree centrality (DC) from midsize to large OGs. However, from a technical perspective, these inconsistencies can also stem from the prevalent use of fixed threshold settings in many algorithms. For example, when selecting candidate orthologs for clustering or tree building, a threshold in protein sequence similarity or a statistically significant cutoff value is often applied. Given the diverse evolutionary histories and varying selection pressures on proteins, a one-size-fits-all approach using predetermined parameters may not be suitable. Therefore, there is a call for a strategy that circumvents fixed parameters and accounts for differences in protein selection pressures, which is of utmost importance.

Our solution to this challenge is illustrated in Fig. 2, which clearly differentiates orthologs/inparalogs from distant homologs. Figure 2F, in particular, encapsulates the results of our spectral clustering algorithm applied to 2 million seed proteins. This unsupervised algorithm uniquely uses the distribution of over-represented proteins on a 2D plot, taking the relative distance between proteins as input. Instead of relying on the raw amino acid similarity between the seed and orthologs, it identifies clusters based on the density of protein spots in irregular regions of the plot.

Based on the results in Fig. 2F, we propose a discontinuous protein evolution hypothesis and introduce the Signal Jaccard index (SJI) metric to measure the functional similarity of proteins. The SJI is a departure from traditional methods that use arbitrary thresholds based on sequence similarity or significance values. SJI depends on information from the entire gene repertoire across species, like the bidirectional best hit (BBH) method. However, instead of directly identifying 1:1 gene pairs, we split the process into two steps. First, we distinguish signal from noise, establishing a 1:n relationship between a seed protein and its ortholog candidates. Then, we use SJI to measure the overlap of "n" signals between two seeds to indicate their similarity. This two-step process yields a network that integrates the biological information of single proteins into a broader context, with the network's topology reflecting the proteins' evolutionary and functional information.

Networks are common tools in orthology prediction, as exemplified by resources such as eggNOG [9] and OrthoMCL [46]. However, the edges in these networks either rely on species choices or fail to account for the variable selection pressures exerted on different proteins, as reflected by high or low sequence similarity within different protein families on the same network. In contrast, the SJI metric offers a more objective approach since it is unaffected by the selection pressures on different protein families and the species chosen for comparison.

Building on the SJI metric, we also propose the use of DC as a scoring system to assess the reliability of protein orthologs. This approach helps predict protein functions by considering both protein sequence similarity and the broader genomic context of the protein. The biological functions of proteins can often be diverse, exhibiting characteristics such as promiscuity [47], moonlighting [48], or fuzziness [49], which present challenges to the conventional use of ortholog databases. We suggest that these "unusual" functional behaviors can be further explored using different centrality measures within our network.

We also found that many proteins with low DC or from small OGs, often located at the network periphery, are less well annotated, falling into the category known as the "dark matter" of the proteome [50, 51]. Our estimates suggest that approximately 40–50% of all proteins are low DC proteins (OG size < 20 or DC < 0.2). As we incorporate more genomes into our analyses, we anticipate that the core structures identified in this study will remain stable, but the number of peripheral proteins may increase.

## Conclusions

In summary, this work presents several significant conclusions:

1. We propose an unsupervised ortholog prediction algorithm complemented by a novel metric, the Signal Jaccard Index (SJI). This innovative tool provides a new method to assess protein similarity, eschewing reliance on arbitrarily preset parameters. Crucially, it can discern the varied selection pressures exerted on different proteins, reflected by varied average protein sequence similarity within different protein families.
2. We underscore that the primary sources of inconsistency in extant ortholog databases lie in the peripheral nodes of our network, characterized by proteins from small OGs and proteins with low DC scores.
3. The application of DC offers two-fold benefits. It first helps identify proteins in peripheral structures, which warrant extra scrutiny in their ortholog prediction. Subsequently, DC sidesteps the need for intricate operations to ascertain intersections from various ortholog databases. This mitigates the potential arbitrariness arising from the choice of parameters used to determine a consensus, thus facilitating the derivation of "high-quality" orthologs.
4. As a score encapsulating ortholog confidence, we demonstrate that DC maintains a high level of stability, remaining impervious to species choices and exhibiting virtually no arbitrariness. We, therefore, advocate for the incorporation of DC into a scoring system for ortholog prediction benchmarking.

Collectively, our findings present an innovative approach to ortholog prediction. By appreciating the unique characteristics and evolutionary trajectories of proteins, we progress beyond the limitations of arbitrary, universal thresholds. The network we have established offers a quantitative platform for probing diverse aspects of protein evolution, promising to catalyze further research in this arena.

## Supplementary Information

Additional file 1.

Additional file 2.

Additional file3.

Additional file 4.

Additional file 5.

Additional file 6.

Additional file 7.

Additional file 8.

Additional file 9.

## Data Availability

The datasets generated and/or analyzed during the current study are available at www.protdc.org. Scripts used in this work can be found at https://github.com/FangLabNYU/protdc. The human olfactory receptor 52K1, featured in Fig. 2A, has the accession number Q8NGK4. Annotations are available at https://www.uniprot.org/uniprotkb/Q8NGK4/. Additional 2D plots can be accessed at https://www.protdc.org/%5Eprotein_info/9606/Q8NGK4/$. E. coli *ugpE*, also featured in Fig. 2A, has the accession number P10906. Annotations are available at https://www.uniprot.org/uniprotkb/P10906/. 2D plots in our database can be accessed at https://www.protdc.org/%5Eprotein_info/PRJNA225/b3451/$.
