# Supplementary material for: A metric and its derived protein network for evaluation of ortholog database inconsistency
Source: BMC Bioinformatics. 2025 Jan 7;26:6. doi: 10.1186/s12859-024-06023-x (PMC11707888; doi:10.1186/s12859-024-06023-x)
Supplement: Supplementary file 4 — Additional file 4. [file 12859_2024_6023_MOESM4_ESM.pdf]

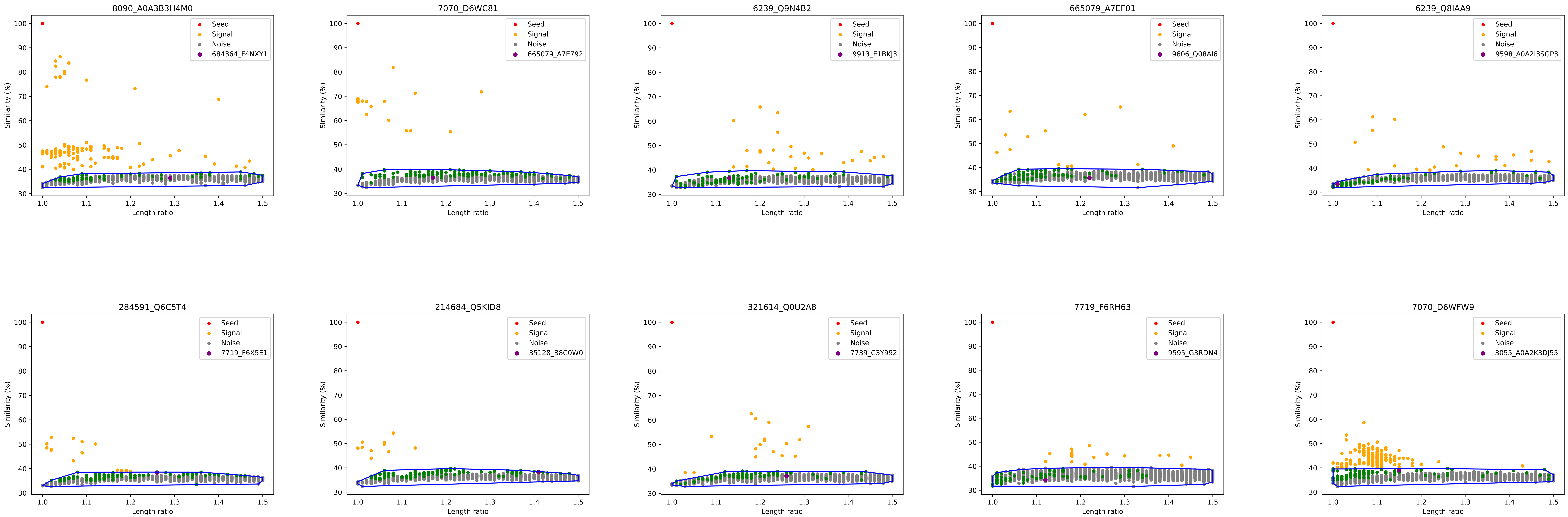

Supplementary Figure 1. Ten examples illustrating TreeFam-A seed-noise pairs and noises exhibiting significant E-values (<10^-6)

Each panel title concatenates the NCBI taxonomy ID and the protein UniProt AC number with an underscore. The pink spot within the noise cloud indicates an ortholog pair formation with the seed, as designated by TreeFam-A. Green spots within the noise cloud represent instances with significant seed-noise E-values of less than 10^-6.
