# Supplementary material for: A metric and its derived protein network for evaluation of ortholog database inconsistency
Source: BMC Bioinformatics. 2025 Jan 7;26:6. doi: 10.1186/s12859-024-06023-x (PMC11707888; doi:10.1186/s12859-024-06023-x)
Supplement: Supplementary file 5 — Additional file 5. [file 12859_2024_6023_MOESM5_ESM.pdf]

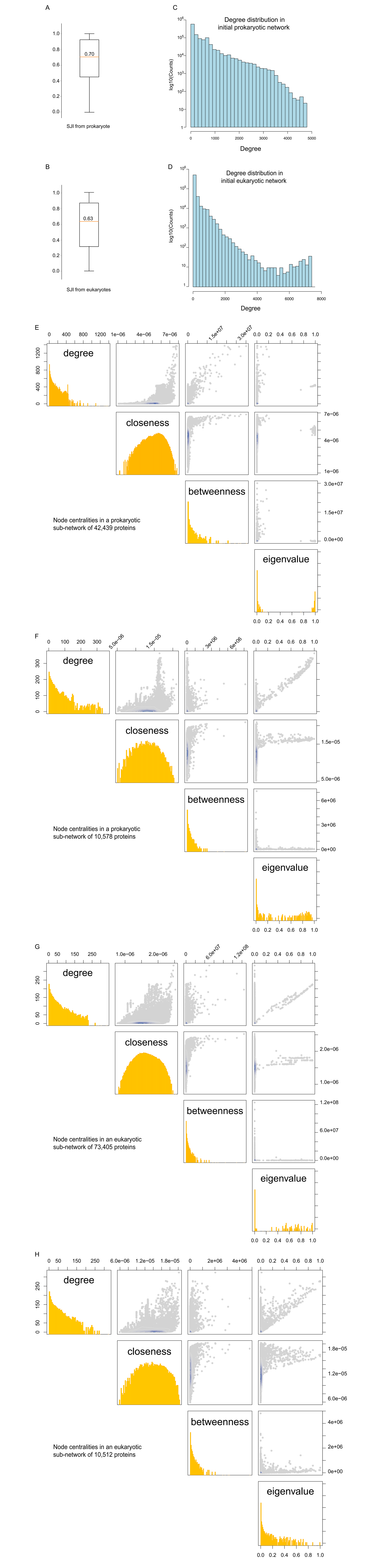

Supplementary Figure 2. Quantitative overview of SJI and the initial network

This figure provides a detailed numerical examination of the Signal Jaccard Index (SJI) and the structure of the initial network. Panels A and B display the boxplots of SJI for prokaryotic and eukaryotic seeds, respectively. Panels C and D illustrate the degree distribution across the entire network. Panels E through H demonstrate correlations between four representative centrality measures - degree, closeness, betweenness, and eigenvector - within four significant representative sub-networks. Due to the extensive size of the initial network, these correlations are most meaningful when examined within local communities or neighboring structures. Yellow histograms in panels E-H reveal the distributions of the four centralities within the sub-networks, while grey scatter plots overlaid with blue density contours present their interrelations.
