# Supplementary material for: A metric and its derived protein network for evaluation of ortholog database inconsistency
Source: BMC Bioinformatics. 2025 Jan 7;26:6. doi: 10.1186/s12859-024-06023-x (PMC11707888; doi:10.1186/s12859-024-06023-x)
Supplement: Supplementary file 6 — Additional file 6. [file 12859_2024_6023_MOESM6_ESM.pdf]

Distribution of bootstrapping correlation coefficient

Eukaryotic

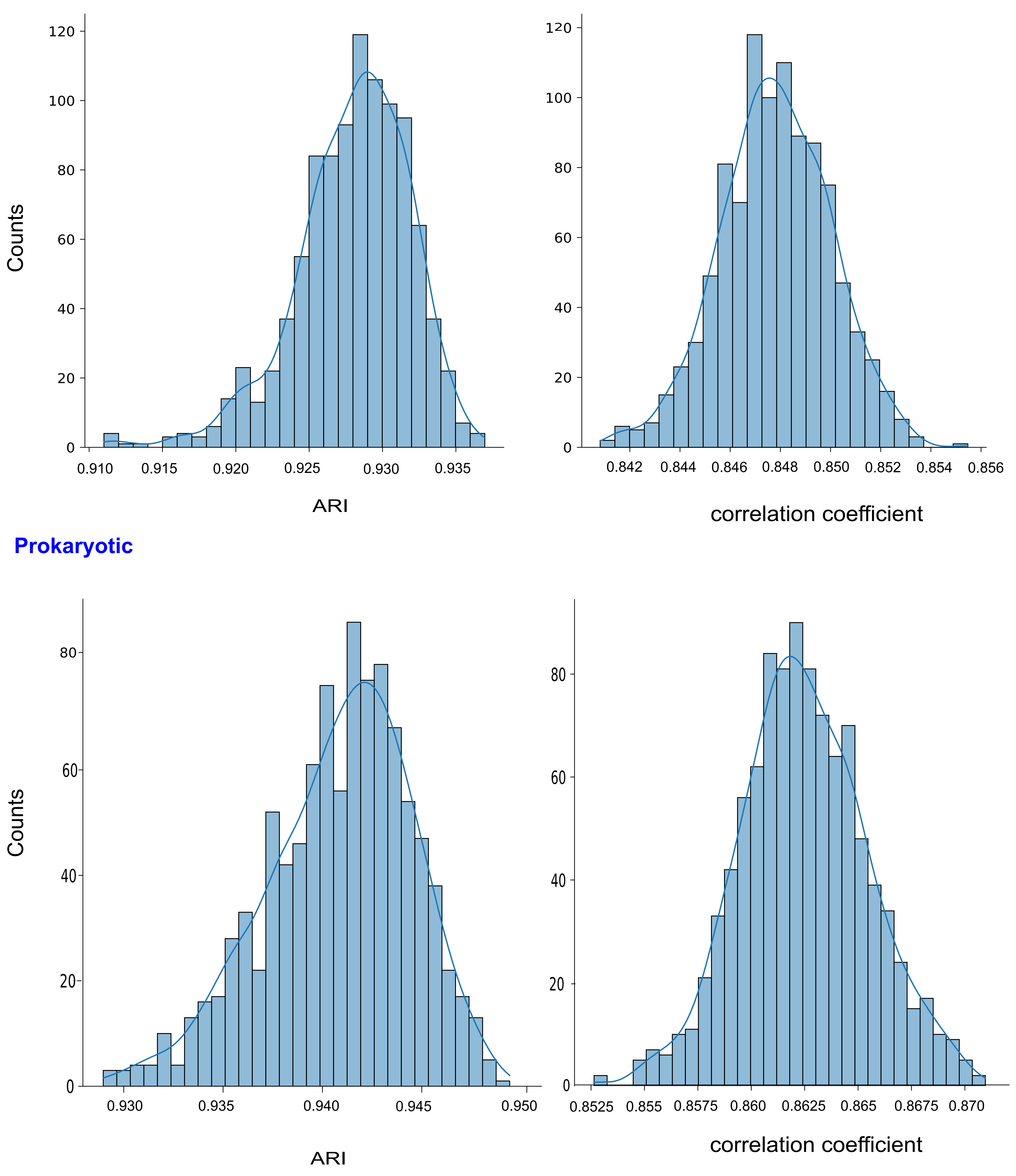

Supplementary Figure 3. Bootstrap analysis of ARI and DC.

Each bootstrap iteration involved the removal of 10% of proteins, with a total of 1000 iterations performed. The left panel depicts the correlation between ARI values computed from bootstrap iterations and the original OGs. The right panel displays the distribution of protein degree centrality correlations derived from these bootstrap iterations.
