# Supplementary material for: A metric and its derived protein network for evaluation of ortholog database inconsistency
Source: BMC Bioinformatics. 2025 Jan 7;26:6. doi: 10.1186/s12859-024-06023-x (PMC11707888; doi:10.1186/s12859-024-06023-x)

Challenge name: TreeFam-A - Agreement with Reference Gene Phylogenies: TreeFam-A

NO CLASSIFICATION

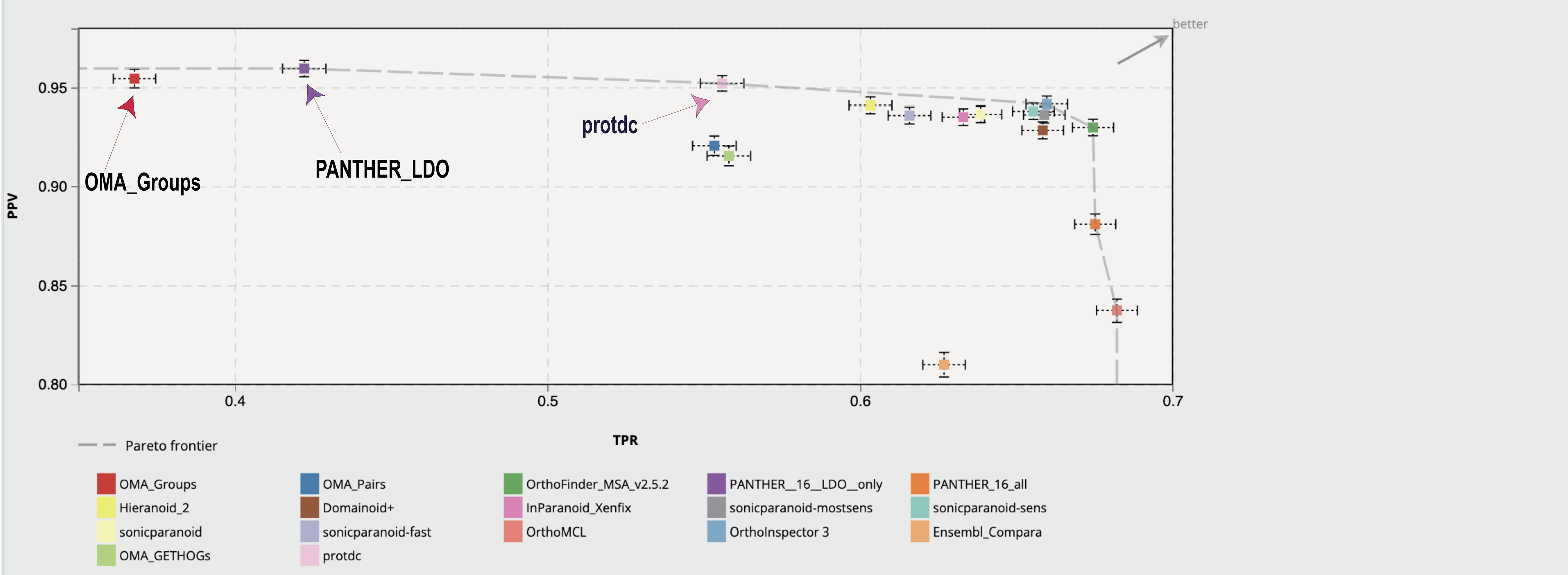

Challenge name: STD_Eukaryota - Species Tree Discordance Benchmark - Eukaryota

NO CLASSIFICATION

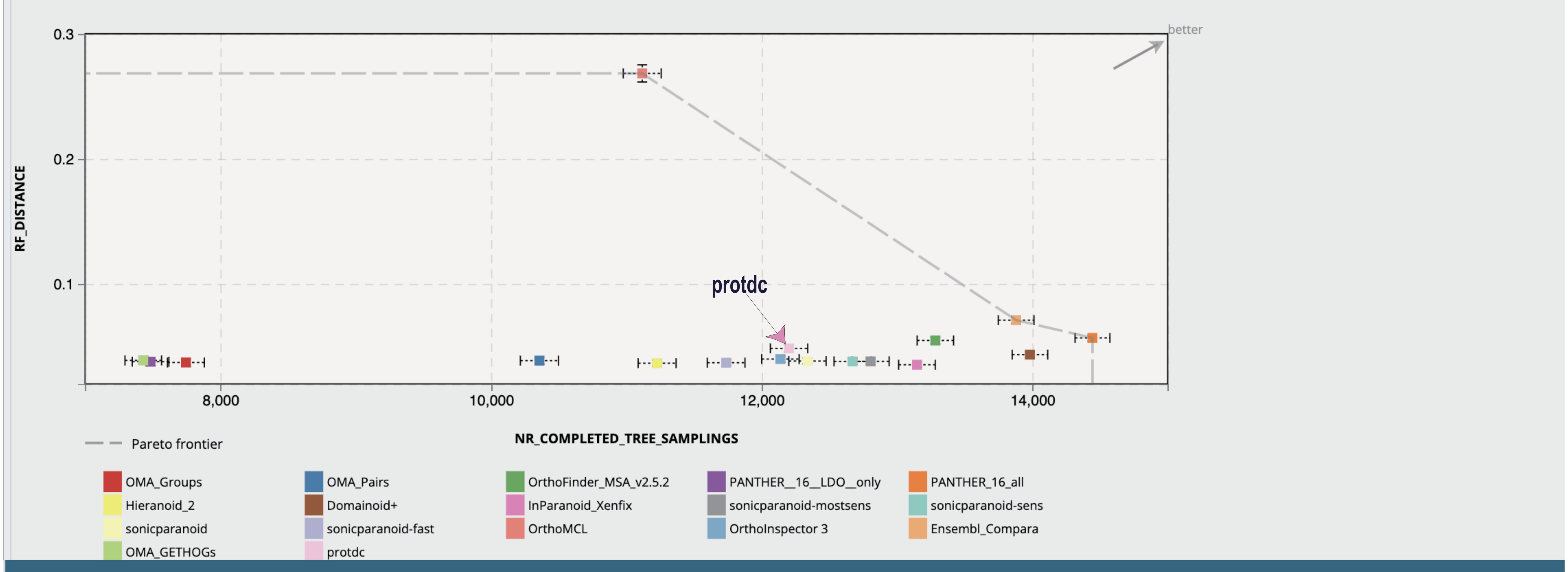

Challenge name: GO - Gene Ontology conservation test

NO CLASSIFICATION

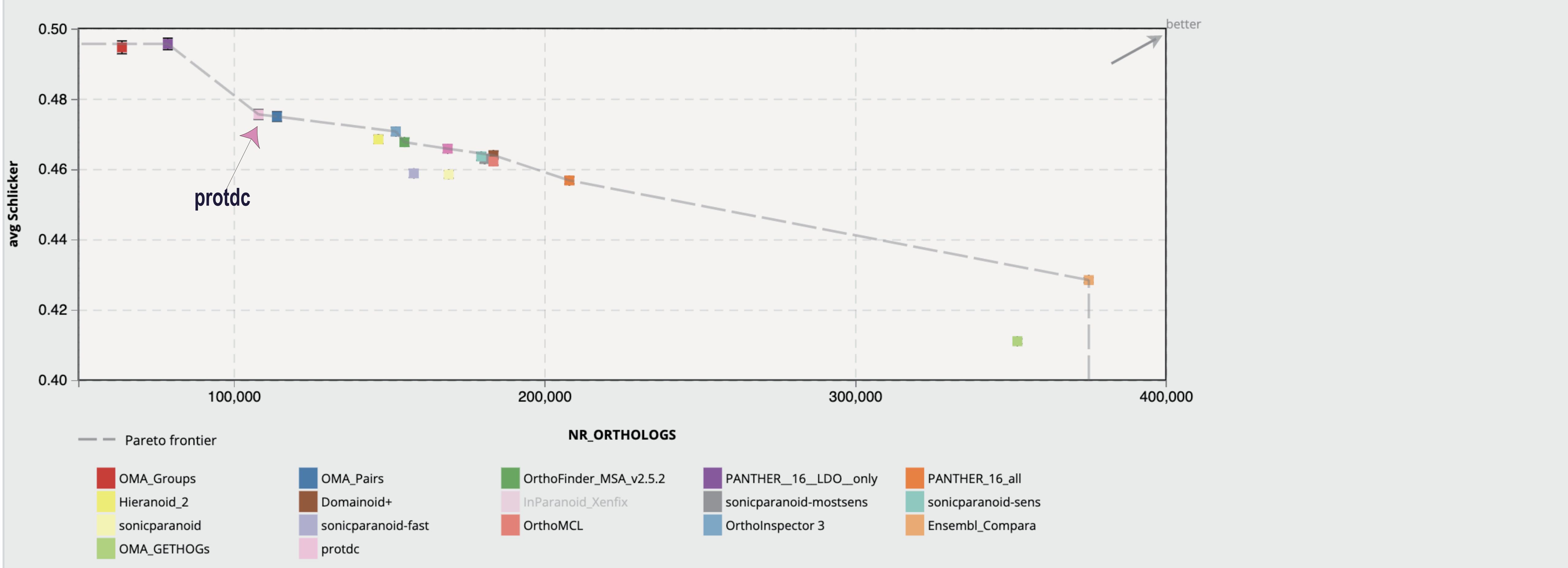

Challenge name: EC - Enzyme Classification (EC) conservation test

NO CLASSIFICATION

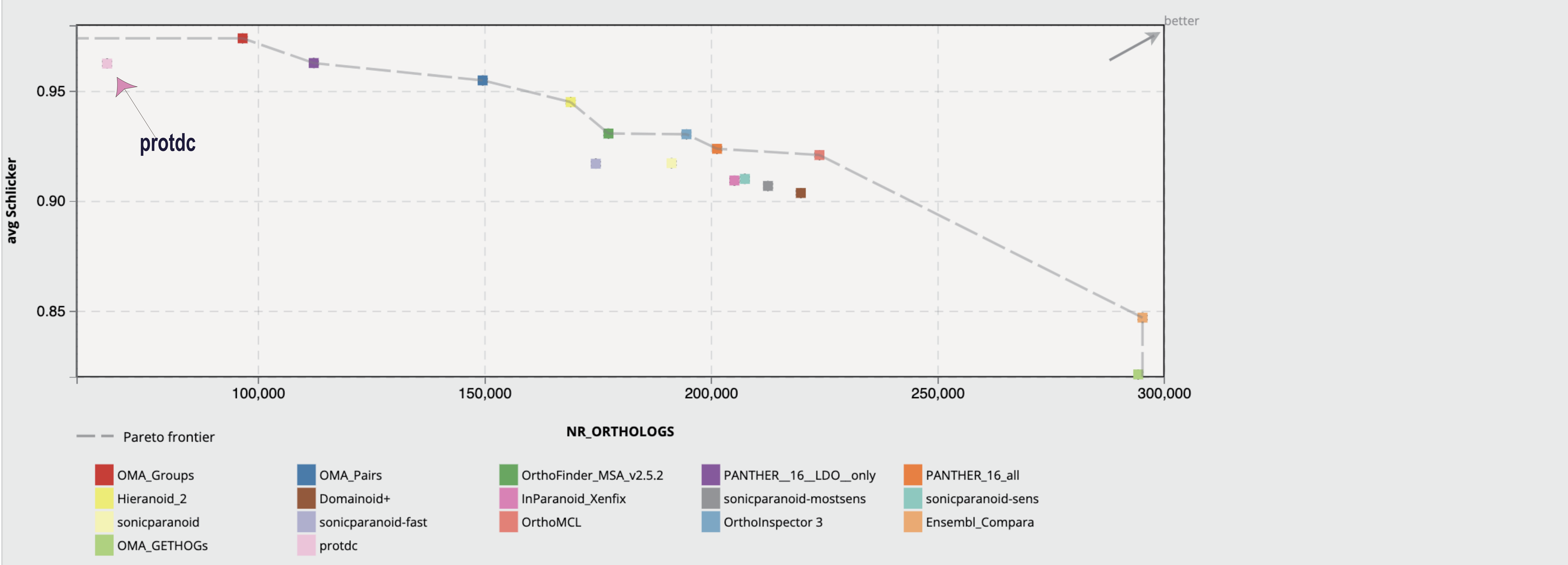

Challenge name: G_STD2_Eukaryota - Generalized Species Tree Discordance Benchmark (Variant 2) - Eukaryota

NO CLASSIFICATION

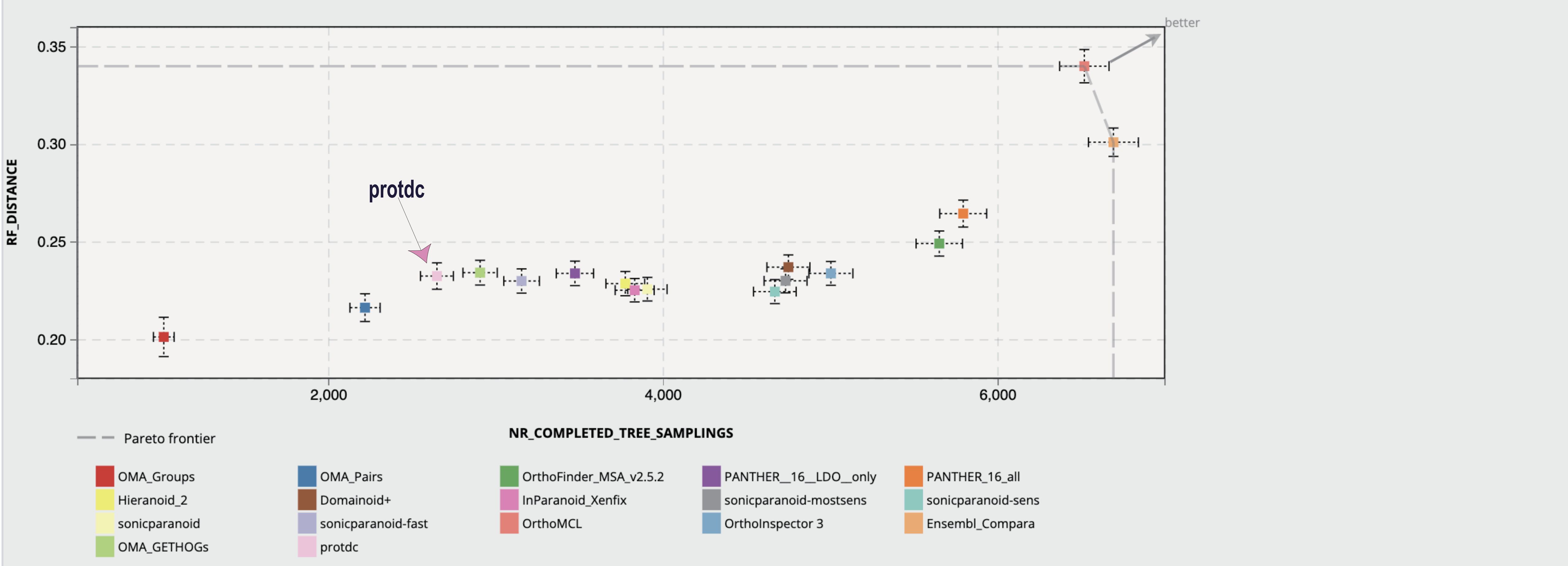

Challenge name: VGNC__2D-plot__TPR_plus_PPV

NO CLASSIFICATION

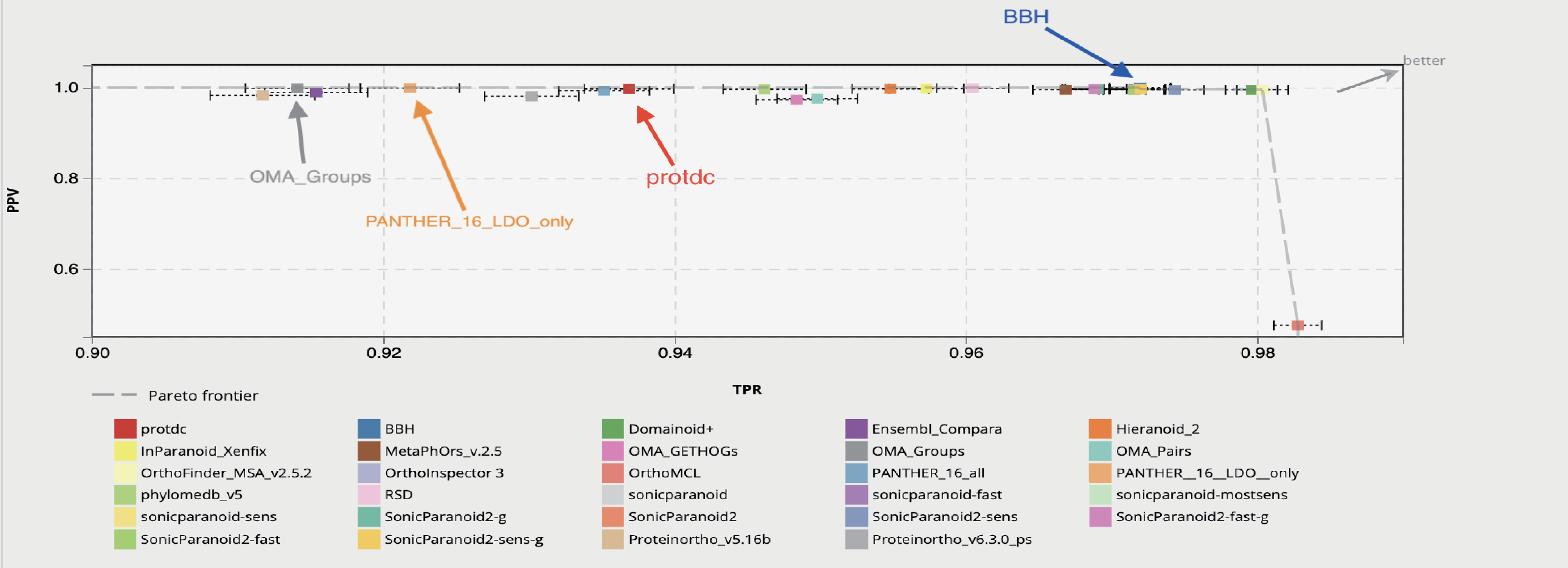

Supplement: Supplementary file 7 — Additional file 7. [file 12859_2024_6023_MOESM7_ESM.pdf]
