# Supplementary material for: A metric and its derived protein network for evaluation of ortholog database inconsistency
Source: BMC Bioinformatics. 2025 Jan 7;26:6. doi: 10.1186/s12859-024-06023-x (PMC11707888; doi:10.1186/s12859-024-06023-x)
Supplement: Supplementary file 8 — Additional file 8. [file 12859_2024_6023_MOESM8_ESM.pdf]

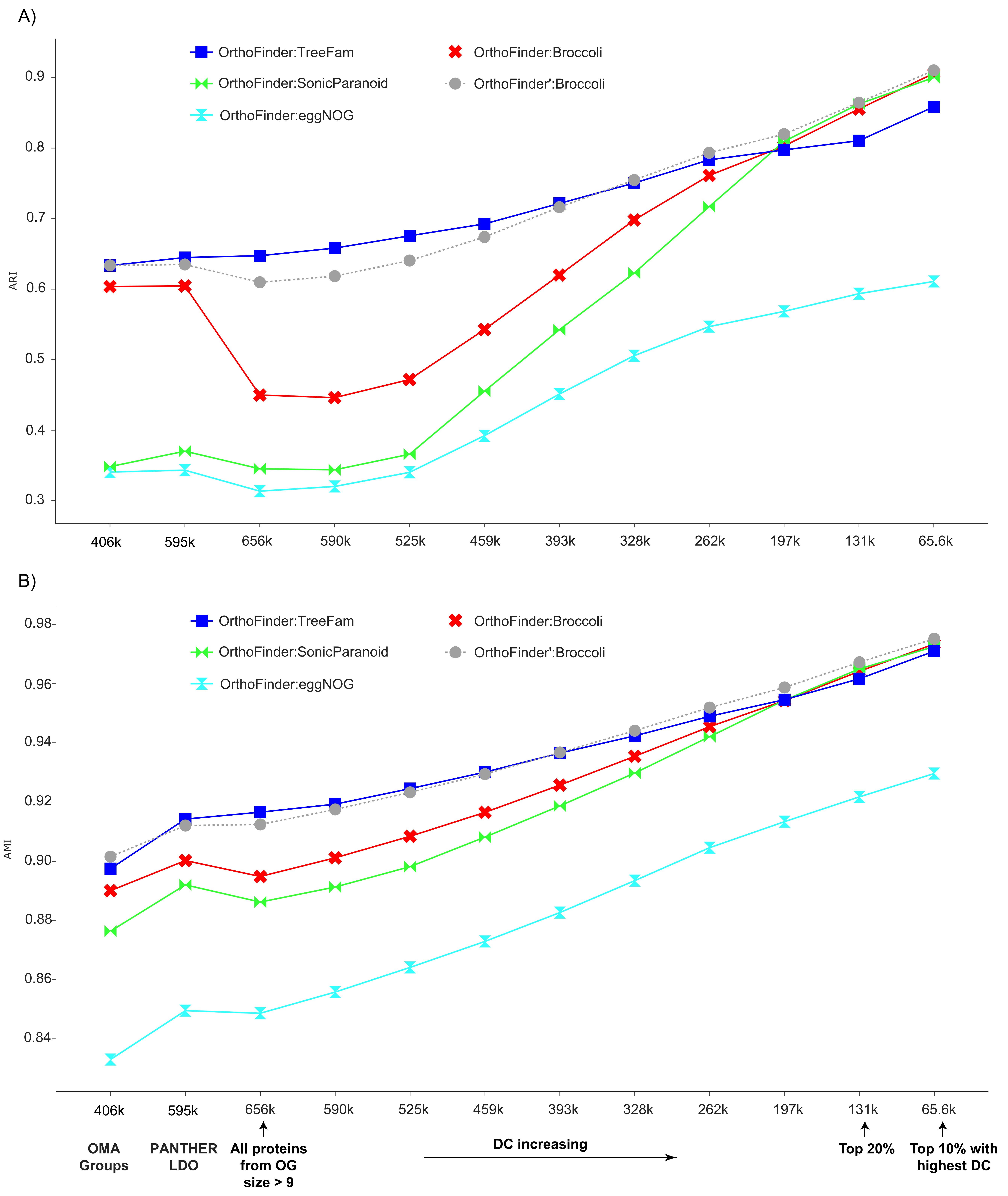

C) Single-Species Inparalogs-Only Ortholog Group (SSIO OG) *

	Number of SSIO OG	% of SSIO OG	Number of Proteins in SSIO OG	% of proteins in SSIO OG
OrthoFinder	25418	47.00%	139881	17.99%
OrthoDB	6823	20.85%	23304	6.40%
eggNOG	4978	18.77%	17907	3.86%
TreeFam	832	8.10%	3328	2.75%
Broccoli	0	0%	0	0%
SonicParanoid	0	0%	0	0%
ProtDC-This Study	15189	28.40%	76919	9.59%

Supplementary Figure 5. Influence of Single-Species Inparalogs-Only Ortholog Groups (SSIOOGs) on ortholog consistency
This figure presents an ortholog concordance analysis influenced by SSIOOGs, with specific emphasis on OrthoFinder's dataset. SSIOOGs refer to ortholog groups (OGs) composed solely of inparalogs (more than one protein) from a single species.

Panels A and B display pairwise ortholog database comparisons, analogous to those in Figure 4 of the main text, computed using ARI and AMI, respectively. Notably, we have supplemented a grey curve that illustrates the comparison between OrthoFinder and Broccoli when SSIOOG proteins are excluded. Panel C offers a summary of SSIOOG data. Since ortholog groups from OrthoFinder, OrthoDB, eggNOG, and TreeFam were extracted from broader datasets, including species that go beyond the 48 eukaryotic proteomes referenced by QfO, their SSIOOGs exhibit substantial differences, potentially reflecting variability introduced by different species inputs when applying various algorithms. On the other hand, Broccoli and SonicParanoid ortholog groups, computed using the 48 QfO reference proteomes, do not contain SSIOOGs. Therefore, it is not appropriate to directly compare OrthoFinder and Broccoli when SSIOOGs are included.

When evaluating our proteins' concordance with other databases, using PANTHER's LDO and OMA Groups as reference baselines, we observed decreased alignment between OrthoFinder and other ortholog databases, particularly between OrthoFinder and Broccoli (the red curve in Panel A). This discrepancy is attributed to the presence of SSIOOGs in OrthoFinder's dataset. Specifically, among the 557K proteins shared between our dataset and OrthoFinder, 28K proteins belong to OrthoFinder's SSIOOGs. After these 28K (5%) proteins are removed, the ARI improves from 0.450 to 0.610 (the 3rd point of the grey curve). AMI appears to be less sensitive to SSIOOGs (Panel B).

It's important to clarify that our intention is not to suggest the inclusion of SSIOOGs in OrthoFinder is problematic. Rather, we highlight the need for careful consideration of SSIOOGs during ortholog comparisons, as they can significantly influence concordance measures. Our own OGs also contain SSIOOGs (Panel C), and the ARI between our 656K proteins and OrthoFinder's 777K proteins is 0.500.
