# Supplementary material for: A metric and its derived protein network for evaluation of ortholog database inconsistency
Source: BMC Bioinformatics. 2025 Jan 7;26:6. doi: 10.1186/s12859-024-06023-x (PMC11707888; doi:10.1186/s12859-024-06023-x)
Supplement: Supplementary file 9 — Additional file 9. [file 12859_2024_6023_MOESM9_ESM.pdf]

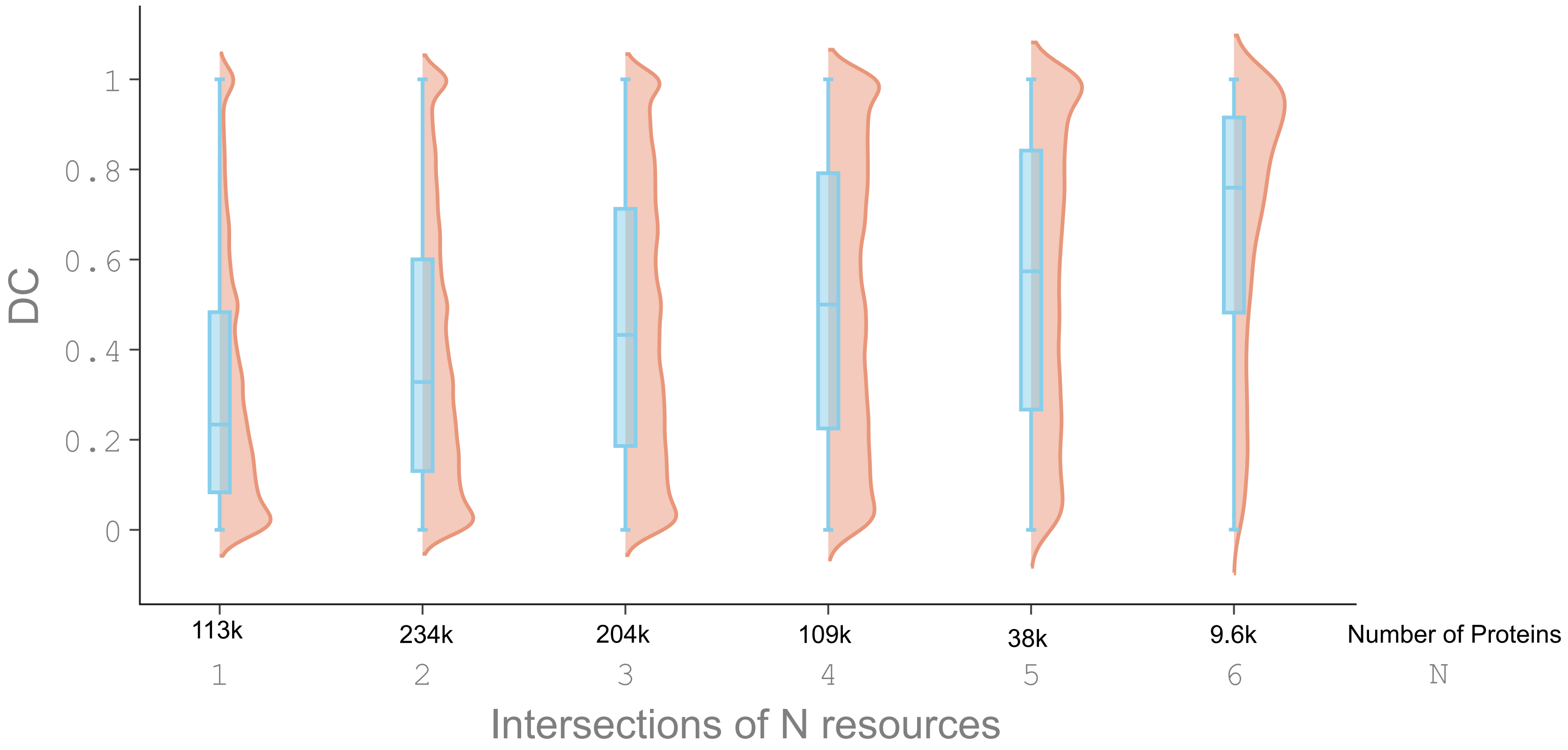

Supplementary Figure 6. Intersections of various ortholog databases versus protein DC

Eukaryotic proteins from the QfO v2021 dataset were extracted from six representative databases: TreeFam, eggNOG, Broccoli, OrthoFinder, OrthoDB, and SonicParanoid. The x-axis signifies the number of databases with consistent ortholog assignments. For instance, the rightmost bin includes only 9K proteins with a unanimous orthology assignment across all six databases. In contrast, the leftmost bin contains 113K proteins with no orthology consensus among the six databases. Meanwhile, 234K, 204K, 109K, and 38K proteins have orthology assignments consistently endorsed by 2, 3, 4, and 5 databases, respectively endorsing their OG assignment.
The y-axis shows the degree centrality distribution for proteins in the corresponding bin. While intersections among these six databases underscore inconsistency in orthology assignments, degree centrality exhibits a weak positive correlation with their concordance trend.
